# Induction of Labor: A Narrative Review for Clinical Use

**DOI:** 10.7759/cureus.94205

**Published:** 2025-10-09

**Authors:** Esther Namutosi, Prosper Akankwasa, Joshua B Matokota, Daniel D Ssempanyi, John Katongole, Jackson Kakooza, Catherine Lewis, Emmanuel Okurut, Daniel U Owu, Nightingale Senaji

**Affiliations:** 1 Department of Obstetrics and Gynecology, Kampala International University, Ishaka, UGA; 2 Department of Surgery, Kampala International University, Ishaka, UGA; 3 Department of Surgery, St. Joseph's Hospital Kitovu, Masaka, UGA; 4 Department of Physiology, Kampala International University, Ishaka, UGA; 5 Department of Obstetrics and Gynecology, Dagoretti Sub-county Hospital Mutuini, Nairobi, KEN

**Keywords:** bishop score, cervical ripening, fetal outcomes, induction of labor, maternal outcomes, oxytocin, prostaglandins

## Abstract

Induction of labor (IOL) is a pivotal obstetric intervention employed when continuing pregnancy poses greater risks than delivery. Cervical readiness, defined as vaginal delivery within 24-48 hours without significant complications, is assessed via the Bishop Score or transvaginal ultrasonography (TVS). TVS is a key predictor of IOL success. Mechanical methods, such as a Foley catheter, and pharmacological agents (e.g., misoprostol and dinoprostone), facilitate cervical ripening. Indications for IOL include postterm pregnancy, hypertensive disorders, and intrauterine fetal demise, with timing tailored to maternal and fetal factors such as 39 weeks for low-risk cases and 37 weeks for mild preeclampsia. Special conditions, such as previous cesarean sections, require individualized approaches due to risks like uterine rupture, while hypertensive disorders necessitate prompt intervention to optimize outcomes. Complications such as failed induction, uterine hyperstimulation, and fetal distress underscore the need for vigilant monitoring. Despite challenges, IOL remains integral to obstetric care, enhancing maternal and neonatal safety through evidence-based practices. Future research should refine success metrics and optimize combined mechanical-pharmacological strategies to improve efficacy and reduce adverse events, ensuring a balanced risk-benefit profile across diverse clinical contexts. This narrative review synthesizes current evidence on IOL, encompassing definitions, indications, methods, clinical scenarios, complications, and outcomes, emphasizing collaborative decision-making between healthcare providers and pregnant individuals.

## Introduction and background

Induction of labor (IOL) is the medical process of starting uterine contractions before they begin naturally, usually with medications or physical methods, to deliver the baby safely [1]. It is a critical obstetric intervention initiated when continuing a pregnancy poses greater risks to maternal or fetal health than delivery, such as in postterm pregnancy (pregnancy extending beyond 41-42 weeks), prelabor rupture of membranes (PROM), or hypertensive disorders of pregnancy [2,3]. The Bishop score, a clinical score used to measure cervical readiness for labor, is frequently employed to predict the likelihood of success [4]. While IOL can prevent serious complications such as stillbirth, eclampsia, and maternal sepsis, it also carries risks, including failed induction, cesarean delivery, uterine hyperstimulation, and fetal distress [5].

Over recent decades, IOL prevalence has increased globally, driven by evolving clinical guidelines, rising maternal age, and improved access to healthcare. In high-income countries, approximately 20%-30% of term pregnancies involve IOL, with rates reported at 22.7% in the United States, 31.6% in the United Kingdom, and up to 35% in Australia [6,7]. In low- and middle-income countries (LMICs), IOL rates are lower, typically ranging from 6% to 15%, largely due to limited healthcare infrastructure and decreased access to induction agents [8,9]. This stark contrast illustrates inequities in access and outcomes: women in high-resource settings benefit from advanced monitoring and a wider choice of induction methods, whereas women in LMICs face higher risks of complications.

Large, randomized trials have significantly influenced practice, demonstrating that elective IOL at 39 weeks in low-risk, nulliparous women reduces cesarean section rates (18.6% vs. 22.2%) without increasing adverse neonatal outcomes [10]. However, in resource-limited settings, challenges such as inadequate monitoring and higher baseline maternal morbidity contribute to elevated risks of complications, including postpartum hemorrhage (PPH) and neonatal mortality [8], making it difficult to translate such evidence in LMIC contexts.

Despite its widespread use, controversies persist regarding the optimal timing, methods, and monitoring of IOL across different settings. The global rise in IOL underscores the need for a comprehensive, evidence-based synthesis to standardize practices and address outcome variability. This narrative review will examine global evidence on indications, methods, predictors of success, complications, and outcomes of IOL, while highlighting practice disparities between high- and low-resource contexts. By doing so, we aim to refine understanding, inform clinical decision-making, and guide strategies to improve maternal and neonatal outcomes worldwide.

## Review

Indications for IOL

Maternal Indications

There are many maternal indications for IOL, as shown in Table 1. These are conditions that threaten maternal health, necessitating delivery to prevent severe complications, and some are briefly described here. One of such maternal conditions is preeclampsia/eclampsia and Hemolysis, Elevated Liver enzymes, and Low Platelet (HELLP) count syndrome. Severe preeclampsia, eclampsia, or HELLP syndrome warrants IOL at ≥34 weeks or earlier if maternal or fetal stability is compromised, with delivery recommended at term for milder cases. High-quality evidence supports this approach, demonstrating that IOL reduced maternal morbidity (e.g., stroke and renal failure) and neonatal complications [2]. Successful IOL at 34-37 weeks due to severe preeclampsia has been shown to optimize outcomes in resource-limited settings [11].

**Table 1:** Maternal indications for labor induction PROM: prelabor rupture of membrane; HELLP: hemolysis, elevated liver enzymes, and low platelet count; PGE2: prostaglandin E2; GDM: gestational diabetes mellitus

Condition	Recommendation	Evidence quality	Strength
PROM	IOL recommended within 24 hours, oxytocin preferred; PGE2 at 24 hours if no contraindications	High	Strong
Unfavorable cervix	Misoprostol (25-50 μg vaginal) or dinoprostone recommended if no uterine abnormalities	High	Strong
Preeclampsia/eclampsia	IOL recommended if severe or at term; immediate delivery if unstable	High	Strong
HELLP syndrome	IOL recommended if ≥34 weeks or delivery indicated	High	Strong
Placental abruption (stable)	IOL considered if stable; immediate delivery if severe bleeding	Moderate	Weak
Severe antepartum hemorrhage	Immediate IOL indicated if stable; often urgent delivery required	Moderate	Strong
Diabetes (gestational or preexisting)	IOL at 38-40 weeks if uncontrolled/complicated; 41 weeks if uncomplicated GDM	Very low	Weak
Thrombophilia	IOL considered if severe or with complications: timing per clinical judgment	Low	Weak
Renal disease	IOL indicated if severe or deteriorating maternal condition: timing per severity	Low	Weak
Obstetric cholestasis	IOL at 37-38 weeks; earlier (36 weeks) if severe biochemical abnormalities	Moderate	Strong
Severe maternal disease (unresponsive to treatment)	Immediate IOL indicated if not responding to treatment	High	Strong
PROM with Group B Streptococcus	IOL recommended promptly to reduce infection risk	High	Strong
Preterm PROM ≥34 weeks	IOL indicated; MgSO_4_ for neuroprotection if <32 weeks along with steroids/antibiotics	Moderate	Strong
Gestational hypertension	IOL indicated if severe or at term (per clinical judgment)	Moderate	Strong

Another maternal indication for IOL is chronic and gestational hypertension. In chronic hypertension, particularly when superimposed with gestational hypertension or severe features, IOL is indicated at 37-39 weeks for controlled cases or ≤34 weeks for severe, uncontrolled cases. This strategy has the advantage of reducing cesarean rates and maternal complications, supported by moderate-quality evidence [12]. In addition, gestational diabetes mellitus (GDM) and preexisting diabetes mellitus require IOL at 38-40 weeks if poorly controlled or complicated (e.g., macrosomia and polyhydramnios), or at 41 weeks for well-controlled GDM. Studies suggest that IOL at 39 weeks reduces neonatal hypoglycemia and cesarean rates [13], even for uncomplicated cases [6].

PROM is another maternal condition where IOL is required within 24 hours, especially in a full-term pregnancy. It is preferably induced with oxytocin to reduce chorioamnionitis and neonatal infection risks. Immediate IOL in such conditions reduces infection rates and neonatal intensive care unit (NICU) admissions [6].

Fetal Indications

Fetal indications for IOL, as shown in Table 2, focus on conditions compromising fetal health, where delivery prevents adverse outcomes.

**Table 2:** Fetal indications for labor induction IOL: induction of labor; PGE2: prostaglandin E2; FOGSI: Federation of Obstetric and Gynecological Societies of India; SOGC: Society of Obstetricians and Gynecologists of Canada; WHO: World Health Organization; NICE: National Institute for Health and Care Excellence; ACOG: American College of Obstetricians and Gynecologists

Condition	Recommendation	Evidence quality	Strength
Postterm pregnancy (>42 weeks)	IOL recommended at 41 weeks	Low	Weak
Intrauterine fetal demise	Immediate IOL or expectant management; misoprostol (24-28 weeks) or PGE2/oxytocin (>28 weeks)	Moderate	Strong
Intrauterine growth restriction	IOL if severe, ≥34 weeks, or with abnormal Doppler; increased surveillance otherwise	Moderate	Strong
Oligohydramnios	IOL if severe or with fetal compromise, often ≥37 weeks	Moderate	Strong
Twins (uncomplicated)	IOL at 37 weeks (FOGSI/Queensland) or 38 weeks (SOGC); not recommended by WHO/NICE/ACOG	Low	Weak

Postterm pregnancy is any pregnancy that extends beyond 42 weeks. IOL is recommended at 41 weeks (>40 weeks + seven days) to reduce stillbirth risks, which increase beyond 42 weeks. Moderate-quality evidence from a 2020 Cochrane review shows a significant reduction in perinatal mortality (RR, 0.27; 95% CI, 0.08-0.98) with IOL at 41 weeks [6].

Chorioamnionitis necessitates IOL. It is an inflammation of the fetal membranes and amniotic fluid, usually due to a bacterial infection. It can occur before labor, during labor, or after delivery and can be acute, subacute, or chronic [14]. Acute chorioamnionitis necessitates immediate IOL to mitigate maternal sepsis and fetal morbidity, with high-quality evidence underscoring its urgency [15].

Intrauterine growth restriction (IUGR) is an estimated fetal weight less than the 10th percentile via ultrasound. In severe IUGR, especially with abnormal Doppler findings (e.g., absent/reversed end-diastolic flow), IOL is indicated at ≥34 weeks with heightened surveillance [16]. Moderate-quality evidence links timely IOL to reduced neonatal morbidity, such as hypoxic-ischemic encephalopathy [17].

Oligohydramnios is defined as decreased amniotic fluid volume for gestational age [18]. IOL for patients with idiopathic oligohydramnios is recommended at 37 weeks of gestation or beyond [19]. No studies or guidelines provide a universal recommendation for delivery timing in early preterm oligohydramnios (28-33 weeks and six days) due to the need to balance risks of prematurity against risks of continuing the pregnancy. The American College of Obstetricians and Gynecologists (ACOG) and the Society for Maternal-Fetal Medicine emphasize that delivery timing decisions should consider maternal and fetal risks, practice environment, and patient preferences; however, they do not specify a gestational age for early preterm cases [20,21].

Intrauterine fetal demise (IUFD) requires immediate IOL or expectant management based on gestational age and maternal preference. Misoprostol is recommended for 24-28 weeks of gestation, while oxytocin or prostaglandin E2 (PGE2) is preferred if >28 weeks. Moderate-quality evidence confirms the safety and efficacy of these regimens, with a 2024 review highlighting reduced maternal complications with misoprostol [3,22].

Elective Induction

Elective IOL is often performed for nonmedical reasons such as convenience. It is increasingly considered in low-risk pregnancies at ≥39 weeks. Evidence from the landmark A Randomized Trial of Induction Versus Expectant Management (ARRIVE) Trial demonstrated no adverse maternal or neonatal outcomes and potential reductions in cesarean births [10]. This practice aligns with ACOG recommendations, emphasizing 39 weeks as the optimal timing to balance neonatal maturity with minimal stillbirth risk [20]. Elective IOL is particularly appealing in high-income settings, where access to monitoring and induction agents is robust, with approximately 25% of pregnancies in such regions undergoing IOL, including elective cases. The incidence of elective IOL is lower in LMICs [23]. However, the decision requires collaborative discussion between healthcare providers and pregnant individuals, ensuring informed consent and realistic expectations about risks, benefits, and methods. Factors such as cervical readiness (Bishop Score ≥6 or favorable transvaginal ultrasonography (TVS) findings) and maternal characteristics (e.g., multiparity, body mass index (BMI) <30) enhance success rates defined as vaginal delivery within 24-48 hours without complications [4,24]. Despite its benefits, elective IOL increases healthcare costs and may elevate risks such as failed induction (20%-40% in nulliparous women with unripe cervices) or uterine hyperstimulation (1%-5%), necessitating careful patient selection and monitoring [1].

Contraindications and risk stratification

Contraindications to IOL are categorized as absolute or relative based on maternal and fetal risks. Absolute maternal contraindications include prior uterine rupture, classical cesarean incision, or myomectomy, due to significant risks of uterine rupture [10]. Active genital herpes and invasive cervical cancer preclude IOL to prevent neonatal transmission and disease progression, respectively [10,25]. On the other hand, relative contraindications include prior cesarean sections when using PGEs like misoprostol, due to elevated uterine rupture risk, particularly in IUFD [26,27].

Fetal and Pregnancy-Related Contraindications

Fetal and pregnancy-related contraindications include placenta previa and vasa previa, which risk catastrophic hemorrhage, and malpresentations like transverse or footling breech, which hinder safe vaginal delivery [10,26]. Severe fetal heart rate abnormalities (Category III tracings) and monoamniotic twins are contraindications to IOL due to risks of hypoxia and cord entanglement [10].

Preinduction evaluation for IOL

The preinduction evaluation is a cornerstone of safe and effective IOL. The evaluation ensures that the timing, method, and clinical context align to optimize maternal and neonatal outcomes. This narrative review synthesizes evidence from the provided literature, focusing on four critical components of preinduction evaluation: confirming gestational age, assessing fetal well-being, evaluating cervical readiness, and ensuring informed consent through shared decision-making. These steps are essential to mitigate risks, tailor interventions, and foster collaborative care, as supported by contemporary obstetric guidelines and studies.

Confirming Gestational Age for IOL

Accurate confirmation of gestational age is critical for determining the appropriateness and timing of IOL, as it directly impacts fetal maturity and maternal-fetal risk profiles. Gestational age guides decisions by balancing the risks of continuing pregnancy against those of delivery. For instance, postterm pregnancies (>41 weeks) are a common indication for IOL due to increased stillbirth risks beyond 42 weeks, with meta-analyses showing reduced perinatal mortality when IOL is initiated at 41 weeks [6]. In cases of mild preeclampsia, IOL is recommended at 37 weeks, while severe cases may necessitate intervention at or before 34 weeks [28]. Elective IOL in low-risk pregnancies is supported at ≥39 weeks, as demonstrated by the ARRIVE Trial, which showed no adverse outcomes and potential reductions in cesarean rates [10].

Gestational age is best confirmed using early ultrasound (before 20 weeks) or reliable last menstrual period data, ensuring precise dating for timing interventions [20]. Inaccurate dating can lead to iatrogenic prematurity or delayed intervention, particularly in conditions like IUGR or gestational diabetes, where precise timing (e.g., 38-40 weeks for complicated diabetes) is essential [20]. Healthcare professionals prioritize IOL at or after 39 weeks in low-risk cases to optimize neonatal outcomes, underscoring the importance of robust gestational age confirmation to inform evidence-based clinical decisions [1].

Assessing Fetal Well-Being Prior to IOL

It is essential to assess fetal well-being before IOL to confirm if the fetus can tolerate labor and to identify any contraindications. Nonreassuring fetal testing, such as Category III fetal heart rate tracings, contraindicates IOL due to hypoxia risks [23]. Fetal well-being is evaluated using nonstress tests, biophysical profiles, or Doppler velocimetry, particularly in high-risk conditions like IUGR or oligohydramnios [10]. For example, IUGR with abnormal Doppler findings may prompt IOL at ≥34 weeks with intensified monitoring to mitigate adverse outcomes, supported by moderate-quality evidence [23].

Fetal distress, occurring in 10% of IOL cases, may necessitate cesarean delivery if labor tolerance is low, highlighting the need for preinduction assessment [4]. In postterm pregnancies or preterm prelabor rupture of membranes (PPROM), confirming fetal well-being ensures IOL safety, as prolonged gestation or membrane rupture heightens risks of hypoxia or infection [10]. For twins or growth-restricted fetuses, normal fetal heart rate patterns improve IOL success, while severe abnormalities preclude induction [29]. These assessments inform method selection and timing, ensuring IOL proceeds only when fetal resilience is confirmed, reducing complications like fetal distress (2%-10% incidence) or perinatal morbidity [5].

Cervical Readiness for IOL

Cervical readiness is a critical determinant of successful IOL, defined as achieving vaginal delivery within 24-48 hours without significant complications. The Bishop Score and TVS are primary tools for assessing cervical favorability. The Bishop Score evaluates cervical dilatation, consistency, effacement, position, and fetal station, with a score ≥6 indicating a favorable cervix [30]. Scores >6 correlate with a 68.6% vaginal delivery rate compared to 48.5% in failed cases, while unfavorable scores (≤6) are associated with a 29.4% cesarean rate [4]. Higher scores (8-10) strongly predict success, though subjective digital examination introduces interobserver variability [4,31]. TVS provides an objective alternative, measuring cervical length, funneling, and position, with a score ≥6.5 showing high predictive accuracy (sensitivity 99%, specificity 94%, area under the curve (AUC) 0.705) for successful IOL [4].

TVS surpasses the Bishop Score (AUC 0.735), with superior sensitivity (77% vs. 65%) and specificity (93% vs. 86%) at a cutoff of ≥4, and an AUC up to 0.911. Funneling, a key TVS indicator, is linked to shorter delivery times, enhancing its role in guiding induction methods. It reduces variability, making it preferred where available, while the Bishop Score remains effective for rapid bedside assessment [4]. However, TVS requires ultrasound expertise and equipment, limiting its use in resource-constrained settings, while the Bishop Score’s simplicity supports broader applicability [32].

Cervical ripening, facilitated by collagen degradation and hormonal mediators, is augmented by mechanical (e.g., Foley catheter) or pharmacological (e.g., misoprostol) methods when the cervix is unfavorable [32]. The choice of ripening agent is informed by these assessments, with TVS enabling precise interventions in term pregnancies and potentially reducing failed inductions [4].

Informed Consent and Shared Decision-Making for IOL

Informed consent and shared decision-making are essential components of the preinduction process, ensuring that pregnant individuals are actively involved in decisions regarding IOL. Collaborative discussions between healthcare providers and the patient must cover indications, risks, benefits, methods, and realistic expectations, fostering a woman-centered approach [3]. For instance, elective IOL at ≥39 weeks, supported by the ARRIVE trial, requires comprehensive counseling about potential cesarean rate reductions vs. risks such as failed induction (20%-40% in nulliparous women with unripe cervices) or uterine hyperstimulation (1%-5%) [10]. In complex cases, such as previous cesarean sections or IUFD, shared decision-making is crucial due to heightened risks of uterine rupture or emotional considerations [33,34].

Providers must discuss method-specific risks, such as misoprostol’s contraindication in scarred uteri, and alternatives, such as expectant management, tailoring decisions to individual circumstances [22]. Preinduction counseling also addresses maternal factors (e.g., nulliparity, BMI <30) and fetal factors (e.g., birth weight <4 kg) that impact IOL success, empowering women to make informed choices [35,36]. This collaborative process, guided by experienced obstetricians, aligns IOL with clinical needs and patient values, enhancing satisfaction and optimizing outcomes [37].

Methods of labor induction

IOL is a critical obstetric intervention employed to initiate uterine contractions and achieve vaginal delivery when continuing pregnancy poses greater risks than delivery. The choice of induction method, pharmacologic, mechanical, or nonpharmacologic, depends on cervical readiness, clinical indications, and maternal-fetal factors, with the goal of achieving vaginal delivery within 24-48 hours without significant complications [3]. Pharmacologic methods (PGE analogs, oxytocin protocols, and mifepristone), mechanical and physical methods (balloon catheters, membrane sweeping, and amniotomy), and nonpharmacologic and complementary methods (nipple stimulation, acupuncture, and herbal supplements) are all common routes of induction used. We discuss mechanisms, efficacy, safety, and clinical roles, emphasizing evidence-based practices to optimize maternal and neonatal outcomes.

Pharmacologic Methods

Pharmacologic agents play a vital role in IOL by targeting biochemical pathways to ripen the cervix and stimulate uterine contractions. These include PGE analogs (PGE1/misoprostol and PGE2/dinoprostone), oxytocin protocols, and mifepristone, each with distinct mechanisms and clinical applications.

PGE analogs are pivotal for IOL, facilitating cervical ripening and uterine contractions by mimicking endogenous PGEs. Misoprostol, a PGE1 analog, is administered vaginally, orally, or sublingually (25-50 µg every four to six hours) and is highly effective for ripening unripe cervices (Bishop Score <6). It degrades collagen, dilates the cervix, and induces contractions, achieving higher vaginal delivery rates (68.6% vs. 48.5% in failed cases) and shorter delivery times compared to dinoprostone [38]. Misoprostol is particularly effective for indications like postterm pregnancy and PROM, though it risks tachysystole, necessitating careful dosing [4]. The International Federation of Gynecology and Obstetrics (FIGO) recommends misoprostol 25-50 µg every four hours vaginally or every two hours orally for IOL ≥25 weeks, emphasizing its versatility across gestational ages [39].

Dinoprostone (PGE2), available as a gel or vaginal insert, promotes collagen breakdown, vasodilation, and contractions with a controlled onset but slower progression than misoprostol. PGE1 outperforms PGE2 in efficiency, though the controlled release of dinoprostone reduces hyperstimulation risks in some settings [40]. Sequential PGE2-PGE1 protocols enhance vaginal delivery rates [41]. Both agents are contraindicated in women with prior cesarean sections due to uterine rupture risks, with misoprostol posing a greater hazard [22]. TVS (score ≥6.5) improves agent selection by predicting IOL success (sensitivity 99%, specificity 94%, AUC 0.705), optimizing outcomes for PGE-based IOL [4].

Oxytocin is a synthetic hormone that stimulates uterine contractions via oxytocin receptors and serves primarily as an augmentation agent, enhancing labor after cervical ripening. Its standalone use in IOL is limited due to minimal ripening effects [28]. It is typically administered intravenously following mechanical or PGE-induced ripening, particularly when the cervix is favorable (Bishop Score ≥6). It is often combined with methods like Foley catheters or misoprostol to achieve vaginal delivery within 24-48 hours [4]. In a cohort study involving 322 women, 95% of the women had a spontaneous vaginal delivery success with a reduced time (5.42 hours) following IOL with oxytocin compared to other methods, such as 13 hours for dinoprostone, 8.5 hours for misoprostol, and 9.0 hours for the balloon catheter [42].

Oxytocin carries a 1%-5% risk of hyperstimulation, necessitating continuous fetal monitoring and careful dose titration. For PROM, oxytocin is preferred within 24 hours of rupture to expedite delivery [4]. High-dose oxytocin regimens may improve IOL success but increase hyperstimulation risks, highlighting the importance of precise protocol management [35].

Mifepristone, a progesterone receptor antagonist, is used off-label for IOL, particularly in cases of IUFD. It primes the uterus by blocking progesterone, increasing uterine sensitivity to PGEs, and promoting cervical softening [43]. Mifepristone is recommended at 200 mg orally, followed by misoprostol after one to two days for IUFD at ≥13 weeks (e.g., 50-100 µg misoprostol every four hours vaginally or every two hours orally at ≥34 weeks) [44]. Successful mifepristone-misoprostol induction has been reported for second- and third-trimester IUFD, though prolonged induction may occur in complex cases [33]. The combination of mifepristone and misoprostol is safe and effective for IUFD management. Mifepristone’s use in routine IOL remains limited due to regional regulatory variations and insufficient evidence for broader indications; however, its efficacy in special scenarios, such as IUFD, is well-established [44].

Mechanical and Physical Methods

Mechanical methods physically alter the cervix and stimulate endogenous PGE release, offering safer profiles than pharmacologic agents by minimizing uterine hyperstimulation. These include balloon catheters, membrane sweeping, and amniotomy. Each method is tailored to specific cervical states and clinical contexts.

Balloon catheters, such as the Foley catheter and double-balloon catheter, are highly effective for ripening unripe cervices (Bishop Score <6). The Foley catheter, a single-balloon device, is inserted transcervically, inflated with 30-60 mL of saline, and taped to the thigh for tension, mechanically dilating the cervix and triggering PGE release [45].

Liyanapatabandi et al. found that Foley catheters were comparable to misoprostol in achieving vaginal delivery, with lower hyperstimulation risks, making them ideal for women with prior cesarean sections or in resource-limited settings [46]. Double-balloon catheters apply dual pressure above and below the cervix, potentially enhancing ripening, though variable evidence has been reported on superiority over single-balloon designs [47].

Combination strategies, such as a Foley with misoprostol or extraamniotic saline infusion, shorten induction-to-delivery intervals and increase vaginal delivery rates. Higher balloon volumes (60-80 mL) may reduce labor duration in nulliparous women, further optimizing outcomes [48]. Balloon catheters are safe, cost-effective, and versatile. Gupta et al. confirmed equivalent efficacy to PGE2 and better neonatal outcomes [49].

Membrane sweeping involves digital separation of the amniotic membranes from the lower uterine segment, releasing local PGEs to promote cervical ripening and labor onset [50]. Hassan reported increased spontaneous labor and vaginal delivery rates, with reduced complications such as PPH [51]. Its efficacy is modest compared to catheters or PGEs, making it an adjunct or early ripening method for low-risk women nearing spontaneous labor [47]. Membrane sweeping carries minimal risks, though discomfort is common, and its success depends on a partially favorable cervix (Bishop Score ≥4). However, it is relevant in low-risk settings where gradual ripening is feasible [4].

Amniotomy, or artificial rupture of membranes (AROM), involves controlled membrane rupture to release amniotic fluid, enhancing cervical ripening and labor progression when the cervix is accessible and favorable (Bishop Score ≥6). Preethi and Tripathy suggest its use after ripening, as their study focused on intact membranes, positioning AROM as an adjunct method [4]. Dick et al. noted reduced chorioamnionitis rates and improved outcomes in term PROM, underscoring AROM’s role in specific scenarios [52]. AROM complements other methods, boosting labor progression once ripening is underway, but is not suitable for initiating IOL in unripe cervices due to infection risks, especially if labor is delayed [28].

Nonpharmacologic and Complementary Methods

Nonpharmacologic and complementary methods, such as nipple stimulation, acupuncture, and herbal supplements, are less commonly used in clinical practice due to limited evidence but are explored in specific contexts, particularly for low-risk pregnancies or patient-driven preferences. Nipple stimulation, acupuncture, and herbal supplements are not extensively covered in evidence-based guidelines for IOL due to limited robust data. Nipple stimulation, involving manual or pump-based breast stimulation, mimics oxytocin release to induce contractions. Small studies suggest it may reduce postterm pregnancies in low-risk women, but efficacy is inconsistent, and unmonitored use risks hyperstimulation [53]. Acupuncture, targeting specific points to stimulate uterine activity, shows mixed results in small trials for reducing induction-to-delivery time. While generally safe, its efficacy lacks support from large-scale studies [54]. Herbal supplements, such as evening primrose oil or red raspberry leaf, are anecdotally used to soften the cervix, but no high-quality trials confirm efficacy or safety [55]. Safety considerations include the need for monitoring to prevent overstimulation in nipple stimulation, identifying qualified practitioners for acupuncture, and avoiding untested herbal substances. These methods are not recommended as primary IOL strategies but may complement other approaches in low-risk settings with informed consent and close oversight [56].

Predictors of induction success

The success of IOL is influenced by cervical readiness, maternal characteristics, and emerging diagnostic tools, such as ultrasound and biomarkers. These predictors guide method selection and optimize outcomes, reducing the risk of failed induction and associated complications.

Bishop Score Thresholds

The Bishop Score, a clinical tool assessing cervical dilatation, consistency, effacement, position, and fetal station, is a cornerstone for predicting the success of IOL. A score ≥6 indicates a favorable cervix, correlating with higher vaginal delivery rates. Preethi and Tripathy report a 68.6% vaginal delivery rate for Bishop Scores >6 compared to 48.5% in those whose induction failed, reinforcing the predictive value of cervical readiness [4]. Mlodawski et al. further refine this, noting that scores of 8-10 (e.g., dilatation 3-4 cm) yield significantly higher success than scores of 6-7, reflecting greater cervical readiness [31]. Assemie et al. confirm a Bishop Score ≥5 as a critical threshold for improved IOL outcomes, with studies emphasizing the use of cervical ripening agents for scores ≥5 [57].

Influence of Parity, BMI, and Gestational Age

Maternal characteristics, including parity, BMI, and gestational age, significantly influence IOL outcomes. Parity is a critical factor. Preethi and Tripathy found nulliparity prevalent in both successful (60%) and failed (58.8%) IOL groups, with no significant differences [4], but Tesemma et al. report higher failure rates in nulliparous women, particularly those with unfavorable cervices, due to prolonged labor and increased cesarean risk [35]. Habeebullah and Devarasetty observed greater IOL success in multiparous women, whose prior vaginal deliveries enhance cervical remodeling and labor progression [58].

BMI also plays a role. Preethi and Tripathy reported mean BMIs of 25.5-25.8 kg/m² in their cohort [4], while Farah et al. and Habeebullah and Devarasetty associate BMI <30 with higher IOL success, noting that obesity (BMI ≥30) elevates cesarean risk due to prolonged labor and dystocia [29,58]. Gestational age also impacts outcomes variably. In a comparative multicenter study, Tesemma et al. demonstrated higher success rates for induction performed at 40-42 weeks [35], while Farah et al. observed increased vaginal delivery rates when induction occurred closer to spontaneous cervical ripening [29]. These findings highlight the importance of tailored IOL strategies based on maternal characteristics.

Outcomes and complications

IOL outcomes encompass maternal and neonatal health metrics, with complications ranging from failed induction and cesarean delivery to rare but severe events such as uterine rupture. Understanding these outcomes is crucial for optimizing IOL protocols and minimizing risks.

Maternal Outcomes

The goal of IOL is to achieve vaginal delivery within 24-48 hours, a target met in 68.6% of cases when the cervix is favorable (Bishop Score >6) [4]. However, success drops to 48.5% in failed cases, with 29.4% of these women requiring cesarean delivery. This stark contrast underscores the cervix’s pivotal role in IOL outcomes. Adu-Bonsaffoh and Seffah further highlight that IOL elevates cesarean risk compared to spontaneous labor, particularly for nulliparous women and those with hypertensive disorders. The increased surgical intervention in these groups often stems from prolonged labor or failure to progress, amplifying maternal and healthcare burdens [8].

PPH, defined as blood loss ≥500 mL, looms as a significant concern in IOL. The risk escalates with oxytocin use or prolonged labor, both of which are common in induced deliveries. Preethi and Tripathy suggest that failed IOL, with its 29.4% cesarean rate, doubles PPH odds due to surgical intervention, though exact rates remain elusive [4]. Intriguingly, the choice of cervical ripening method can influence this risk. Liyanapatabandi et al. found that mechanical methods may reduce PPH risk compared to PGEs by minimizing uterine hyperstimulation, offering a safer pathway for some women [46].

Infections like chorioamnionitis and endometritis are another hurdle, particularly when labor is protracted or membranes rupture early. PROM with or without leaking, each accounts for 10% of IOL indications. Failed inductions, with their extended labor durations, heighten exposure to these infections, though specific rates are not detailed. A promising development is the use of TVS, which may lower infection risk by reducing the need for invasive vaginal exams compared to traditional Bishop Score assessments [4]. This noninvasive approach could reshape clinical practice, prioritizing maternal safety.

Failed induction, defined as the inability to achieve vaginal delivery within 24-48 hours, is a primary driver of cesarean delivery. Preethi and Tripathy report a 29.4% cesarean rate in failed IOL compared to 19.6% in successful cases, often linked to unfavorable cervices (Bishop Score ≤6) or indications like postterm pregnancy (24%) or preeclampsia (17.3%) [4]. Nulliparity and hypertensive disorders further elevate this risk [8]. Innovations like TVS offer hope by improving patient selection for IOL, potentially reducing failures. Combined mechanical-pharmacological approaches, such as Foley catheters with misoprostol, may also lower cesarean rates by enhancing cervical ripening [46].

Uterine rupture, though occurring in <1% of cases, is a grave complication, particularly for women with scarred uteri from prior cesareans. Cheng et al. [5] identify nulliparity and hypertensive disorders as risk factors. Misoprostol and dinoprostone are contraindicated in women with prior cesareans due to heightened rupture risk, making mechanical methods a safer choice [22,46].

Uterine hyperstimulation, characterized by greater than five contractions in 10 minutes or contractions lasting greater than two minutes, affects 1%-5% of IOL cases, primarily with the use of pharmacological agents such as misoprostol or oxytocin. Hyperstimulation can lead to fetal hypoxia and emergency cesarean sections, posing risks to both mother and neonate. Foley catheters again emerge as safer alternatives, with minimal overstimulation [46]. Meticulous dosing and monitoring are necessary to mitigate these adverse outcomes.

Neonatal Complications

Neonates born via IOL, especially in high-risk pregnancies, face increased risks of NICU admission. IOL for infants with birth weights ≥3.5 kg or gestational ages <41 weeks has been linked to higher NICU rates [5]. These admissions often stem from complications such as respiratory distress or hypoglycemia, notably in GDM cases [13]. Fetal distress is also an indication for IOL, hinting at potential neonatal compromise that may necessitate intensive care [4].

Data on Apgar scores post-IOL are sparse, but fetal distress during induction can lower scores due to hypoxia from hyperstimulation or prolonged labor. Liyanapatabandi et al. suggest that mechanical ripening methods using Foley catheters, with their lower hyperstimulation risk, may support better neonatal outcomes compared to PGEs [46]. Fetal distress itself is a critical concern, reported as an indication in 10% of IOL cases, with rates of 4.6% in failed inductions vs. 8.6% in successful ones [4]. This discrepancy suggests that distress often prompts cesarean delivery. Cheng et al. emphasize that continuous fetal monitoring is essential, as distress is tied to perinatal morbidity, particularly in postterm or growth-restricted fetuses [5].

IOL is a double-edged sword, offering timely delivery but introducing risks that demand careful navigation. Successful vaginal delivery hinges on cervical readiness and patient selection, with innovations like TVS and combined ripening methods showing promise in reducing cesarean rates and complications. Maternal risks like PPH and infection, alongside neonatal challenges such as NICU admissions and fetal distress, highlight the need for tailored approaches. Uterine rupture and hyperstimulation, though infrequent, underscore the importance of method selection and vigilant monitoring. As research advances, integrating noninvasive assessments and safer ripening techniques will be key to enhancing the safety and efficacy of IOL, ensuring better outcomes for mothers and their newborns.

Considerations for special populations

IOL in special populations requires tailored approaches due to unique physiological and clinical challenges that influence success rates, complications, and optimal methods. The following sections detail considerations for women with previous cesarean sections, multiple pregnancies, preterm induction, and those with obesity or advanced maternal age.

Women With Previous Cesarean Section

For women with a prior cesarean section, IOL is a high-stakes endeavor, shadowed by the specter of uterine rupture. Misoprostol and dinoprostone are contraindicated due to their association with heightened rupture risk, particularly in cases of IUFD [22]. Instead, mechanical methods like Foley catheters emerge as safer allies, offering a pathway to vaginal birth after cesarean (VBAC) in carefully selected cases [52]. AROM, which achieves high VBAC rates and shorter induction durations, eases the journey to delivery [52]. Grand multiparous women with scarred uteri may undergo IOL without a significantly increased rupture risk, provided methods are chosen judiciously [59]. The decision to induce should not be taken lightly. Shared decision-making, weighing the <1% rupture risk against indications of preeclampsia or postterm pregnancy, should be emphasized [4]. For women with classical or high transverse incisions, however, IOL remains off-limits, as the risk of rupture looms too large [60].

Multiple Pregnancies

The landscape shifts for women carrying multiple pregnancies, where IOL requires a delicate calibration of timing and technique. Recommendations vary, with some guidelines advocating IOL at 37 weeks for uncomplicated twin pregnancies, while others lean toward 38 weeks, reflecting the paucity of robust data on optimal timing [34]. Noncephalic presentation of the first twin or monoamniotic twins poses absolute barriers to IOL, as risks of cord entanglement and delivery complications soar. Evidence for IOL in twins is limited. However, mechanical methods with Foley catheters are favored to minimize hyperstimulation, a particular concern in multifetal gestations [46]. When indications such as IUGR or preeclampsia arise, IOL may be initiated as early as 34 weeks, paired with heightened fetal surveillance to safeguard both mother and babies [34].

Preterm Induction

Preterm IOL, often prompted by PPROM, severe preeclampsia, or chorioamnionitis, introduces its own set of challenges. In cases of PPROM ≥34 weeks, IOL is recommended, often with magnesium sulfate for neuroprotection if <32 weeks, alongside steroids and antibiotics to bolster fetal resilience. Immediate IOL is critical for chorioamnionitis to curb maternal and fetal morbidity, a strategy backed by strong evidence [34]. Misoprostol 25-50 µg every four hours vaginally is effective, but for preterm cases with prior cesarean sections, mechanical methods are preferred to avoid rupture [22]. The risk of failed induction and cesarean delivery looms large, especially with unripe cervices, making TVS a vital tool for precise cervical assessment [4]. In mild preeclampsia, preterm IOL may reduce neonatal respiratory distress, but severe cases often demand immediate delivery, sometimes before 34 weeks, to avert dire outcomes [29].

Obesity and Advanced Maternal Age

Women with obesity (BMI ≥30 kg/m²) or advanced maternal age (≥35 years) face additional hurdles in IOL, as prolonged labor, higher cesarean rates, and comorbidities complicate the process. A BMI value of <30 kg/m² has been reported to produce a successful outcome of IOL in women of reproductive age [29,36]. Obesity amplifies risks of PPH and infection, particularly in failed inductions, as dystocia often leads to surgical intervention. For older mothers, early IOL at 39 weeks is advocated, especially in pregnancies from assisted reproductive technology, to mitigate stillbirth risk, a strategy supported by trials showing no adverse outcomes [10]. Nulliparity, common among older women, further elevates failure rates with unripe cervices, underscoring the need for enhanced cervical ripening. Combined mechanical-pharmacological approaches, such as Foley catheters with misoprostol, offer promise in these populations [61,62].

Current guidelines and evidence-based practices

Guidelines from ACOG, National Institute for Health and Care Excellence (NICE), WHO, and FIGO provide a framework for IOL, emphasizing evidence-based practices while acknowledging variations in institutional protocols and healthcare access. This section summarizes these guidelines, explores regional practice differences, and discusses cost-effectiveness and access implications.

American College of Obstetricians and Gynecologists

ACOG recommends IOL for postterm pregnancy (>41 weeks), preeclampsia (≥37 weeks for mild, ≤34 weeks for severe), and GDM (38-40 weeks if uncontrolled). It advises against IOL in women with prior classical cesarean section or active genital herpes. Cervical ripening is essential for Bishop Scores <6, with misoprostol (25-50 µg) or Foley catheters preferred [1,20]. ACOG supports IOL at 39 weeks for low-risk pregnancies based on the ARRIVE trial, citing reduced cesarean rates [10]. Continuous fetal monitoring is mandated to manage risks like hyperstimulation (1%-5%) [1,20].

National Institute for Health and Care Excellence

NICE recommends IOL at 41 weeks for postterm pregnancies and within 24 hours for term PROM, using oxytocin or PGE2. It advises against routine IOL before 41 weeks in low-risk cases and cautions against PGEs in prior cesarean deliveries [63]. NICE's 2021 update controversially suggested IOL at 39 weeks for ethnic minority women, raising concerns about evidence strength [64]. For PPROM ≥34 weeks, IOL is indicated, with expectant management preferred <34 weeks unless complications arise [63].

World Health Organization

The WHO endorses IOL for postterm pregnancy (>41 weeks), PROM within 24 hours, and preeclampsia or IUFD. It recommends misoprostol (25-50 µg every four to six hours) or Foley catheters, with oxytocin for augmentation postripening. WHO contraindicates IOL in placenta previa, vasa previa, and prior uterine rupture [3]. The 2020 Labor Care Guide emphasizes woman-centered care and collaborative decision-making, prioritizing TVS or Bishop Score assessment [28].

The International Federation of Gynecology and Obstetrics

FIGO provides detailed misoprostol dosing for IOL (25-50 µg every four hours vaginally or two hours orally for ≥25 weeks), IUFD, and other obstetric indications. It supports mechanical methods for prior cesarean section and endorses IOL at 37-38 weeks for obstetric cholestasis. FIGO emphasizes safety, contraindicates PGEs in scarred uteri, and advocates TVS for cervical assessment [39].

Institutional and Regional Practice Variations

Institutional practices vary based on resource availability, clinician expertise, and regional guidelines. In high-resource settings (e.g., the United States, the United Kingdom), TVS is increasingly integrated due to its predictive accuracy, guiding method selection [4]. However, low-resource settings rely on Bishop Score assessments and mechanical methods due to limited ultrasound access [46]. Regional differences also arise from guideline interpretation. Queensland Health advocates IOL at 37 weeks for twins, contrasting with the WHO's conservative stance [65]. In Ghana, Adu-Bonsaffoh and Seffah documented that there is a high cesarean rate with IOL due to nulliparity and hypertensive disorders, reflecting resource-constrained monitoring [8]. Cultural factors also influence the practice of IOL, and there is no consensus over NICE's ethnic-specific recommendations, which some clinicians resist due to insufficient evidence [64].

Cost-Effectiveness and Healthcare Access Implications

Cost-effectiveness is a critical consideration, particularly in low-resource settings. Saunders et al. found that synthetic hygroscopic cervical dilators (SHCDs) reduce hospital costs and cesarean rates compared to PGEs, requiring minimal staff oversight [66]. Foley catheters are cost-effective in resource-limited settings, with Liyanapatabandi et al. noting that their safety and efficacy are comparable to those of misoprostol [46]. However, misoprostol and dinoprostone increase costs due to monitoring for hyperstimulation [67]. TVS, while accurate, requires ultrasound equipment and trained personnel, limiting its feasibility in low-income regions [4]. Access disparities exacerbate outcomes of IOL. For instance, in Somaliland, Farah et al. reported low IOL success in obese women due to inadequate facilities, while high-resource settings benefit from combined mechanical-pharmacological protocols [29]. Addressing these gaps requires scalable solutions, such as SHCDs and training programs, to enhance the use of mechanical methods.

Research gaps and future directions

Innovations in Cervical Ripening

Current cervical ripening methods, such as Foley catheters, misoprostol, and dinoprostone, are effective but face limitations, including variable success rates and risks of hyperstimulation [1,4]. Emerging research highlights novel agents, such as nitric oxide donors (e.g., isosorbide mononitrate), which enhance metalloproteinase (MMP) activity with minimal contractile risks. However, their slower labor progression necessitates further trials to optimize dosing and combination strategies [68]. Combined mechanical-pharmacological approaches, such as Foley catheter use with misoprostol or extraamniotic saline infusion, show promise in reducing induction-to-delivery intervals but lack standardized protocols [48]. Future research should explore biomaterials, such as hyaluronic acid-based gels and targeted cytokine therapies to mimic natural ripening processes, potentially improving efficacy and safety across diverse cervical states.

Role of Ultrasound

TVS has emerged as a superior predictor of IOL success compared to the Bishop Score, providing objective measurements of cervical length, funneling, funnel width, position, and distance to the external os. Preethi and Tripathy conducted a prospective study of 150 term singleton pregnancies, finding a TVS score ≥6.5 highly predictive of successful IOL (sensitivity, 99%; specificity, 94%; AUC, 0.705; p < 0.01), with funneling associated with shorter delivery times [4]. Bajpai et al. report TVS's superior AUC (0.907 vs. 0.815 for Bishop Score) and higher sensitivity (77% vs. 65%) and specificity (93% vs. 86%) at a cutoff of ≥4, underscoring its precision in detecting cervical ripening changes [69]. TVS's objectivity reduces variability, although its predictive values (positive predictive value, 52.6%; negative predictive value, 49%) indicate limitations in ruling out IOL failure, and its application requires ultrasound expertise [4].

Biomarkers

Beyond ultrasound and clinical scoring, emerging proteomic and cytokine biomarkers are being explored to predict induction success. For instance, cervicovaginal fluid proteomics has identified protein signatures associated with spontaneous labor onset, while cervicovaginal cytokine concentrations have been shown to correlate with impending labor [70]. MMPs (MMP-8, MMP-9), interleukin-6, and fetal fibronectin also show promise for enhancing IOL prediction [37,71]. Novel cervical ripening strategies, including nitric oxide donors and hyaluronic acid gels, are also under evaluation to enhance efficacy and safety across different settings [71]. These biomarkers could complement TVS by quantifying biochemical changes, improving patient selection and timing. However, clinical implementation remains limited, requiring further validation to establish standardized thresholds and routine use.

Longitudinal Studies on Long-Term Outcomes

Most IOL studies focus on immediate outcomes, such as vaginal delivery within 24-48 hours or cesarean rates, with limited data on long-term maternal and neonatal effects [4]. Complications like PPH, chorioamnionitis, and NICU admissions are well-documented, but their impact on maternal mental health, pelvic floor integrity, and neonatal development remains underexplored [1,5]. Elective IOL at 39 weeks, supported by the ARRIVE Trial, reduces cesarean rates but lacks follow-up on childhood outcomes [10]. Longitudinal cohort studies are needed to assess these effects, particularly in women with GDM and previous cesarean section cases [13,34].

Standardizing Induction Protocols Globally

Global variations in IOL practices, ranging from TVS-guided protocols in high-resource settings to reliance on Foley catheters in low-resource areas, contribute to outcome disparities [4,46]. Guidelines from ACOG, WHO, NICE, and FIGO differ in timing and agent preferences [1,3,39]. These inconsistencies complicate care in resource-limited settings, where cesarean rates and morbidity are higher [8]. Future efforts should focus on harmonizing protocols through international consensus, prioritizing cost-effective methods such as Foley catheters and SHCDs, and addressing training gaps to ensure equitable access to safe IOL [66].

## Conclusions

IOL is an important part of modern obstetric care, especially in managing conditions such as postterm pregnancy, preeclampsia, and gestational diabetes. When the cervix is favorable, vaginal delivery is safe, successful, and generally preferred. Providing safe and effective induction depends on careful assessment, effective communication with the patient, and teamwork among healthcare providers. There is a need for future research to create consistent protocols, discover better ways to prepare the cervix, and understand the long-term effects of induction on both mothers and babies.

## References

[1] (2009). ACOG practice bulletin no. 107: induction of labor. Obstet Gynecol.

[2] Magee LA, Smith GN, Bloch C (2022). Guideline no. 426: hypertensive disorders of pregnancy: diagnosis, prediction, prevention, and management. J Obstet Gynaecol Can.

[3] World Health Organization (2022). WHO recommendations on induction of labour, at or beyond term. WHO recommendations on induction of labour, at or beyond term.

[4] Preethi RU, Tripathy S (2024). Comparison of sonographic score and Bishop score in the prediction of successful labor induction in term patients: a prospective observational study. Clin Epidemiol Glob Health.

[5] Cheng TS, Zahir F, Carolin SV (2024). Risk factors for labour induction and augmentation: a multicentre prospective cohort study in India. Lancet Reg Health Southeast Asia.

[6] Middleton P, Shepherd E, Morris J, Crowther CA, Gomersall JC (2020). Induction of labour at or beyond 37 weeks' gestation. Cochrane Database Syst Rev.

[7] Seijmonsbergen-Schermers AE, Peters LL, Goodarzi B (2020). Which level of risk justifies routine induction of labor for healthy women?. Sex Reprod Healthc.

[8] Adu-Bonsaffoh K, Seffah J (2022). Factors associated with adverse obstetric events following induction of labour: a retrospective study in a tertiary hospital in Ghana. Afr Health Sci.

[9] Vogel JP, Souza JP, Gülmezoglu AM (2013). Patterns and outcomes of induction of labour in Africa and Asia: a secondary analysis of the WHO Global Survey on Maternal and Neonatal Health. PLoS One.

[10] Grobman WA, Rice MM, Reddy UM (2018). Labor induction versus expectant management in low-risk nulliparous women. N Engl J Med.

[11] Chatzakis C, Liberis A, Zavlanos A, Petousis S, Tsakmaki E, Dinas K, Sotiriadis A (2021). Early delivery or expectant management for late preterm preeclampsia: a meta-analysis of randomized controlled trials. Acta Obstet Gynecol Scand.

[12] Magee LA, Kirkham K, Tohill S (2024). Determining optimal timing of birth for women with chronic or gestational hypertension at term: the WILL (When to Induce Labour to Limit risk in pregnancy hypertension) randomised trial. PLoS Med.

[13] Zielińska M, Feduniw S, Kajdy A, Żmuda E, Baran A, Rabijewski M (2022). 377 risk of adverse perinatal outcome after labor induction in pregnancies complicated by gestational diabetes. Eur J Obstet Gynecol Reprod Biol.

[14] Fowler JR, Simon LV (2025). Chorioamnionitis. StatPearls [Internet].

[15] Lukanović D, Batkoska M, Kavšek G, Druškovič M (2023). Clinical chorioamnionitis: where do we stand now?. Front Med (Lausanne).

[16] Lausman A, Kingdom J (2021). How and when to recommend delivery of a growth-restricted fetus: a review. Best Pract Res Clin Obstet Gynaecol.

[17] Sammaritano LR (2020). Antiphospholipid syndrome. Best Pract Res Clin Rheumatol.

[18] Bynarowicz T, Jenkins SM, Shanks AL (2025). Oligohydramnios. StatPearls [Internet].

[19] Alam K, Rizwan A, Hussain AI, Farooq S, Iftikhar T, Mehdi M (2023). The fetomaternal outcome of induction of labour in idiopathic oligohydramnios. Ann PIMS.

[20] American College of Obstetricians and Gynecologists’ Committee on Obstetric Practice (2021). Medically indicated late-preterm and early-term deliveries: ACOG Committee opinion, number 831. Obstet Gynecol.

[21] Spong CY (2015). Protocol 44: indicated late‐preterm and early‐term deliveries. Protocols for High‐Risk Pregnancies.

[22] Sanchez-Ramos L, Levine LD, Sciscione AC (2024). Methods for the induction of labor: efficacy and safety. Am J Obstet Gynecol.

[23] Tsakiridis I, Mamopoulos A, Athanasiadis A, Dagklis T (2020). Induction of labor: an overview of guidelines. Obstet Gynecol Surv.

[24] Venkatanarayanan N, Walker KF (2021). Evidence around early induction of labor in women of advanced maternal age and those using assisted reproductive technology. Best Pract Res Clin Obstet Gynaecol.

[25] Viteri OA, Sibai BM (2018). Challenges and limitations of clinical trials on labor induction: a review of the literature. AJP Rep.

[26] Nggada BJ (2022). Induction of labour. Current Challenges in Childbirth.

[27] Matega TH, Maindo M-AA, Bosenge J-DN (2021). Labor induction with oral misoprostol in term pregnancy: a clinical trial. J Biosci Med (Irvine).

[28] Mitchell JM, Nolan C, El Shaikh M, Cullinane S, Borlase D (2023). National clinical practice guideline: induction of labour. October.

[29] Farah FQ, Aynalem GL, Seyoum AT, Gedef GM (2023). The prevalence and associated factors of success of labor induction in Hargeisa maternity hospitals, Hargeisa Somaliland 2022: a hospital-based cross-sectional study. BMC Pregnancy Childbirth.

[30] Elghorori MR, Hassan I, Dartey W, Abdel-Aziz E (2006). A way to lend objectivity to Bishop score. J Obstet Gynaecol.

[31] Mlodawski J, Mlodawska M, Plusajska J, Detka K, Bialek K, Swiercz G (2023). Repeatability and reproducibility of potential ultrasonographic Bishop score parameters. J Clin Med.

[32] Vince K, Matijević R (2022). Comparison of intracervical and intravaginal prostaglandin E2 for induction of labor in term pregnancies with unfavorable cervix: randomized controlled trial. Eur J Obstet Gynecol Reprod Biol.

[33] Beshar I, Thomson K, Byrne J (2021). Misoprostol-augmented induction of labour for third trimester fetal demise in a patient with prior hysterotomies. BMJ Case Rep.

[34] Agarwal S, D'Souza R, Dy J (2022). Induction of labour in patients with prior caesarean births or uterine surgery. Best Pract Res Clin Obstet Gynaecol.

[35] Tesemma MG, Sori DA, Gemeda DH (2020). High dose and low dose oxytocin regimens as determinants of successful labor induction: a multicenter comparative study. BMC Pregnancy Childbirth.

[36] Devarasetty S, Habeebullah S (2019). Maternal factors affecting outcome of induction of labour. Int J Reprod Contracept Obstet Gynecol.

[37] Marconi AM (2019). Recent advances in the induction of labor. F1000Res.

[38] Mendez-Figueroa H, Bicocca MJ, Gupta M, Wagner SM, Chauhan SP (2021). Labor induction with prostaglandin E(1) versus E(2): a comparison of outcomes. J Perinatol.

[39] FIGO Mifepristone & Misoprostol and Misoprostol Only Dosing Charts 2023. (2023 (2025). FIGO mifepristone & misoprostol and misoprostol only dosing charts 2023. https://www.figo.org/figo-mifepristone-misoprostol-and-misoprostol-only-dosing-charts-2023.

[40] Socha MW, Flis W, Pietrus M, Wartęga M (2023). Results of induction of labor with prostaglandins E1 and E2 (the RIPE study): a real-world data analysis of obstetrical effectiveness and clinical outcomes of pharmacological induction of labor with vaginal inserts. Pharmaceuticals (Basel).

[41] Puliyath G, Balakrishnan A, Vinod L, Hameed H (2019). Outcome of induction of labor with prostaglandin E1 25 mg vaginal tablet - a retrospective study. Trop J Obstet Gynaecol.

[42] Erasto E, Manguzu MA, Nyondo GG, Kilonzi M, Marealle AI, Mutagonda RF (2025). Safety and effectiveness of different modes of labor induction among pregnant women delivering at referral hospitals in Dar es Salaam, Tanzania: a cohort study. BMC Pregnancy Childbirth.

[43] Safdar EA, Tabassum R, Khan PA, Safdar NA (2023). Cross sectional retrospective study on mifepristone and misoprostol combination vs misoprostol alone for induction of labour in management of IUFD. Acta Pharma Reports.

[44] Hoda A, Ali M, Kulsoom UK (2022). A prospective comparative study of mifepristone and misoprostol vs misoprostol alone for induction of labor in intrauterine fetal death. J South Asian Fed Obstet Gynecol.

[45] Yang F, Huang S, Long Y, Huang L (2018). Double-balloon versus single-balloon catheter for cervical ripening and labor induction: a systematic review and meta-analysis. J Obstet Gynaecol Res.

[46] Liyanapatabandi D, Karunasinghe J, Ziard MH, Prathapan R, Wickramasinghe SI (2021). Pre-induction cervical ripening using a transcervical Foley catheter combined with ISMN vaginal tablets for vaginal birth after previous caesarean section (VBAC)-a comparative study. Open J Obstet Gynecol.

[47] Robinson D, Campbell K, Hobson SR, MacDonald WK, Sawchuck D, Wagner B (2023). Guideline no. 432b: cervical ripening. J Obstet Gynaecol Can.

[48] Priya MJ, Vasantha Kumar S, Nandini S (2023). Induction of labour using transcervical Foley’s catheter with extra amniotic saline infusion versus intracervical prostaglandin E2 gel at term gestation: a comparative study. Int J Reprod Contracept Obstet Gynecol.

[49] Gupta JK, Maher A, Stubbs C, Brocklehurst P, Daniels JP, Hardy P (2022). A randomized trial of synthetic osmotic cervical dilator for induction of labor vs dinoprostone vaginal insert. Am J Obstet Gynecol MFM.

[50] Salau JO, Onile TG, Musa AO, Gbejegbe EH, Adewole AA, Olorunfemi GO, Olumodeji AM (2022). Effectiveness and safety of membrane sweeping in the prevention of post-term pregnancy: a randomised controlled trial. J Obstet Gynaecol.

[51] Hassan AM (2023). Membrane sweeping to induce labor in post-term pregnant women: success rate and outcomes. Cureus.

[52] Dick A, Gutman-Ido E, Chill HH, Karavani G, Ryvkin I, Porat S, Rosenbloom JI (2022). Artificial rupture of membranes as a mode for induction of labor in women with a previous cesarean section- a retrospective cohort study. BMC Pregnancy Childbirth.

[53] Kavanagh J, Kelly AJ, Thomas J (2005). Breast stimulation for cervical ripening and induction of labour. Cochrane Database Syst Rev.

[54] Smith CA, Armour M, Dahlen HG (2017). Acupuncture or acupressure for induction of labour. Cochrane Database Syst Rev.

[55] Vu A (2019). Navigating the myths and truths behind pharmacological drug and herbal supplement use: a guide for pregnant women. Regis University Student Publications (comprehensive collection).

[56] Zamawe C, King C, Jennings HM, Mandiwa C, Fottrell E (2018). Effectiveness and safety of herbal medicines for induction of labour: a systematic review and meta-analysis. BMJ Open.

[57] Assemie MA, Mihiret GT, Mekonnen C, Petrucka P, Getaneh T, Ashebir W (2023). Outcomes and associated factors of induction of labor in East Gojjam Zone, Northwest Ethiopia: a multicenter cross-sectional study. Obstet Gynecol Int.

[58] Habeebullah S, Devarasetty S (2019). Induction of labor: a review. SBV J Basic Clin Appl Health Sci.

[59] Hochler H, Wainstock T, Lipschuetz M, Sheiner E, Ezra Y, Yagel S, Walfisch A (2020). Grandmultiparity, maternal age, and the risk for uterine rupture-a multicenter cohort study. Acta Obstet Gynecol Scand.

[60] Wood S, Cooper S, Ross S (2014). Does induction of labour increase the risk of caesarean section? A systematic review and meta-analysis of trials in women with intact membranes. BJOG.

[61] Ferdausi M, Rahman A, Geeti S, Shoma Chockroborty S, Zaman N, Siddika A (2023). Effectiveness of combined use of misoprostol with intracervical catheter for induction of labour: a randomized control trial. Int J Reprod Contracept Obstet Gynecol.

[62] Al-Rawaf SA, Mousa ET (2024). Comparison of vaginal misoprostol alone versus a combination of vaginal misoprostol and intracervical Foley catheter for inducing labor. J Obstet Gynecol Cancer Res.

[63] (2025). National Institute for Health and Care Excellence. Guideline: inducing labour-draft for consultation. https://www.nice.org.uk/guidance/gid-ng10082/documents/draft-guideline-2.

[64] Mahase E (2021). Doctors question NICE recommendation to induce labour at 39 weeks in ethnic minority women. BMJ.

[65] Queensland Clinical Guidelines (2025). Queensland clinical guidelines. Induction of labour. Guideline no. MN22.22-V10-R27. Induction of labour. Guideline No. MN22.22-V10-R27..

[66] Saunders SJ, Grisamore JL, Wong T, Torrejon Torres R, Saunders R, Einerson B (2022). Moving preinduction cervical ripening to a lower acuity inpatient setting using the synthetic hygroscopic cervical dilator: a cost-consequence analysis for the United States. J Med Econ.

[67] Blagodarniy GV, Mozgovaya EV (2017). Efficacy and safety of labor induction methods using prostaglandin E1. J Obstet Women's Dis.

[68] Jakub M, Marta M, Jagoda G, Kamila G, Stanislaw G (2020). Is unfavourable cervix prior to labor induction risk for adverse obstetrical outcome in time of universal ripening agents usage? Single center retrospective observational study. J Pregnancy.

[69] Bajpai N, Bhakta R, Kumar P, Rai L, Hebbar S (2015). Manipal cervical scoring system by transvaginal ultrasound in predicting successful labour induction. J Clin Diagn Res.

[70] Parry S, Leite R, Esplin MS (2020). Cervicovaginal fluid proteomic analysis to identify potential biomarkers for preterm birth. Am J Obstet Gynecol.

[71] Socha MW, Flis W, Pietrus M, Wartęga M, Stankiewicz M (2022). Signaling pathways regulating human cervical ripening in preterm and term delivery. Cells.

